# Droplet Digital PCR Improves Detection of *BRCA1/2* Copy Number Variants in Advanced Prostate Cancer

**DOI:** 10.3390/ijms26146904

**Published:** 2025-07-18

**Authors:** Phetploy Rungkamoltip, Natthapon Khongcharoen, Natakorn Nokchan, Zaukir Bostan Ali, Mooktapa Plikomol, Tanan Bejrananda, Sarayuth Boonchai, Sarawut Chamnina, Waritorn Srakhao, Pasarat Khongkow

**Affiliations:** 1 Department of Biomedical Sciences and Biomedical Engineering, Faculty of Medicine, Prince of Songkla University, Songkhla 90110, Thailand; 2 Translational Medicine Research Center, Faculty of Medicine, Prince of Songkla University, Songkhla 90110, Thailand; 3 Urology Unit, Department of Surgery, Faculty of Medicine, Prince of Songkla University, Songkhla 90110, Thailand

**Keywords:** droplet digital PCR (ddPCR), *BRCA1*, *BRCA2*, copy number variants (CNVs), MLPA, advanced prostate cancer

## Abstract

*BRCA1* and *BRCA2* are associated with advanced prostate cancer progression and poor prognosis. Copy number variants (CNVs) of these genes play a crucial role in guiding targeted treatments, particularly for patients receiving PARP inhibitors. However, CNV detection using multiplex ligation-dependent probe amplification (MLPA) is often limited by tumor heterogeneity, leading to ambiguous results. This study therefore aimed to evaluate *BRCA1/2* CNVs in advanced prostate cancer patients using droplet digital PCR (ddPCR) and compare the results with MLPA. DNA from 11 advanced prostate cancer tissues was analyzed using both methods, in parallel with four cell lines and seven healthy volunteers. Our findings revealed that ddPCR effectively classified normal CNV groups—including normal control cell lines, healthy volunteers, and samples with normal MLPA final ratios—from deletion groups, which included deletion control cell lines, samples with deletion final ratios from MLPA, and cases with previously ambiguous results. Interestingly, two cases involving *BRCA1* and one case involving *BRCA2* exhibited ambiguous results using MLPA; however, ddPCR enabled more precise classification by applying the Youden Index from ROC analysis and identifying optimal cutoff values of 1.35 for *BRCA1* and 1.55 for *BRCA2*. These optimal thresholds allowed ddPCR to effectively reclassify the ambiguous MLPA cases into the deletion group. Overall, ddPCR could offer a more sensitive and reliable approach for CNV detection in heterogeneous tissue samples and demonstrates strong potential as a biomarker tool for guiding targeted therapy in advanced prostate cancer patients. However, further validation in larger cohorts is necessary to optimize cutoff precision, confirm diagnostic performance, and evaluate the full clinical utility of ddPCR.

## 1. Introduction

Advanced prostate cancer (APC) is a biologically heterogeneous disease, with up to 40% of cases progressing to metastasis despite early intervention [1]. A key driver of this progression is the disruption of homologous recombination repair (HRR) genes, particularly *BRCA1* and *BRCA2*, which are essential for DNA double-strand break repair [1,2,3,4]. Alterations in these genes occur in 5–9% of localized and up to 15–19% of metastatic prostate cancers [5,6]. Previous studies have shown that *BRCA* variants correlate with higher Gleason scores (7–10), elevated prostate-specific antigen (PSA) levels, and increased resistance to treatments, such as hormonal therapy and chemotherapy [6]. While most pathogenic *BRCA1/2* alterations are single nucleotide variants (SNVs) or small insertion/deletion, large genomic alteration (LGR)—including copy number variants (CNVs), especially exon-level deletions—also play a significant role in disease progression [7,8]. CNVs, including deletions and duplications in key *BRCA1/2* exon regions, can significantly impact protein structure and function, making these a crucial factor in optimizing treatment strategies in APC. Several exons in the *BRCA1* gene, including exons, 2, 13, 17, 20, and 22, have been associated with the prostate cancer progression [9,10,11,12]. Notably, CNVs deletions involving *BRCA1* exon 22 and *BRCA2* exon 27 have been shown to impair homologous recombination repair, playing a significant pathogenic role in advanced prostate cancer [3,4]. Accurate detection of *BRCA1/2* CNVs is critical, as current clinical guidelines, including the National Comprehensive Cancer Network (NCCN), AUA/SUO, and European associations, recommend both germline and somatic *BRCA1/2* testing to guide therapeutic decisions, particularly for platinum-based therapies and poly(ADP-ribose) polymerase (PARP) inhibitor [10,12,13,14]. Currently, several methods are available for detecting *BRCA1/2* CNVs, including multiplex ligation-dependent probe amplification (MLPA), next-generation sequencing (NGS), and droplet digital PCR (ddPCR), each with its specific advantages and limitations.

MLPA is widely used and considered a gold standard for exon-level CNV detection in *BRCA1/2* genes [9]. The multiplex assay utilizes up to 50 probes targeting specific DNA regions [15,16,17]. Nevertheless, MLPA reveals several challenges, particularly in heterogeneous somatic cancer tissues like APC [9]. These limitations include an inability to detect aberrations at the single-cell level, an inability to detect unknown point mutations, sensitivity to contaminants, and interference from benign polymorphisms near probe ligation sites [9,18]. Additionally, MLPA analysis requires a minimum of 50% tumor cell content in DNA samples (tumor purity) to ensure reliable detection of CNVs, as the presence of normal cells can obscure the presence of aberrant CNVs, leading to ambiguous interpretations due to tumor heterogeneity [16]. Among other detection challenges, MLPA is labor-intensive, costly, and time-consuming, requiring multiple processing steps and extended turnaround times [9]. Given these limitations, we evaluated whether ddPCR could overcome MLPA’s challenges while offering a more efficient, cost-effective, and accurate approach for detecting *BRCA1/2* CNVs in heterogeneous APC tissue samples.

Digital droplet PCR (ddPCR) is a highly effective and reliable method for detecting CNVs deletions or duplications in heterogeneous DNA sample [7,18,19,20]. Unlike traditional PCR methods, ddPCR offers absolute quantification of target molecules, enabling precise detection of *BRCA1/2* CNV deletions with high sensitivity and specificity, even in tumor DNA highly contaminated by normal cells [7,18,19,20]. ddPCR enables the detection of mutation frequencies as low as 0.01% with high sensitivity, without requiring complex bioinformatic analysis. Its capacity to resolve exon-level CNVs in highly heterogeneous samples makes it particularly suitable for APC and other malignancies characterized by high intratumoral heterogeneity, where only a small fraction of tumor cells may harbor *BRCA1/2* alterations [18,20].

This study aims to demonstrate ddPCR as an alternative approach for detecting *BRCA1/2* CNVs in a highly heterogenous tissue sample from advanced prostate cancer patients. Tissue-derived DNA from these patients was first analyzed using the MLPA assay, considered the gold standard for CNV detection. ddPCR was then performed, using both known *BRCA1/2* CNV cell lines and blood-derived DNA from healthy volunteers as controls to establish thresholds for distinguishing between normal and deletion states. Threshold values were determined using the Youden index calculated from receiver operating characteristic (ROC) curve analysis, ensuring optimal sensitivity and specificity. The cutoff values were applied to interpret ambiguous MLPA results, potentially improving treatment decision-making. By directly comparing MLPA and ddPCR, we evaluated the accuracy, efficiency, and potential of ddPCR as a cost-effective and reliable approach for CNVs detection in clinical practice.

## 2. Results

### 2.1. MLPA Indicated the CNVs of BRCA1/2 in Advanced Prostate Cancer

**Figure 1:**
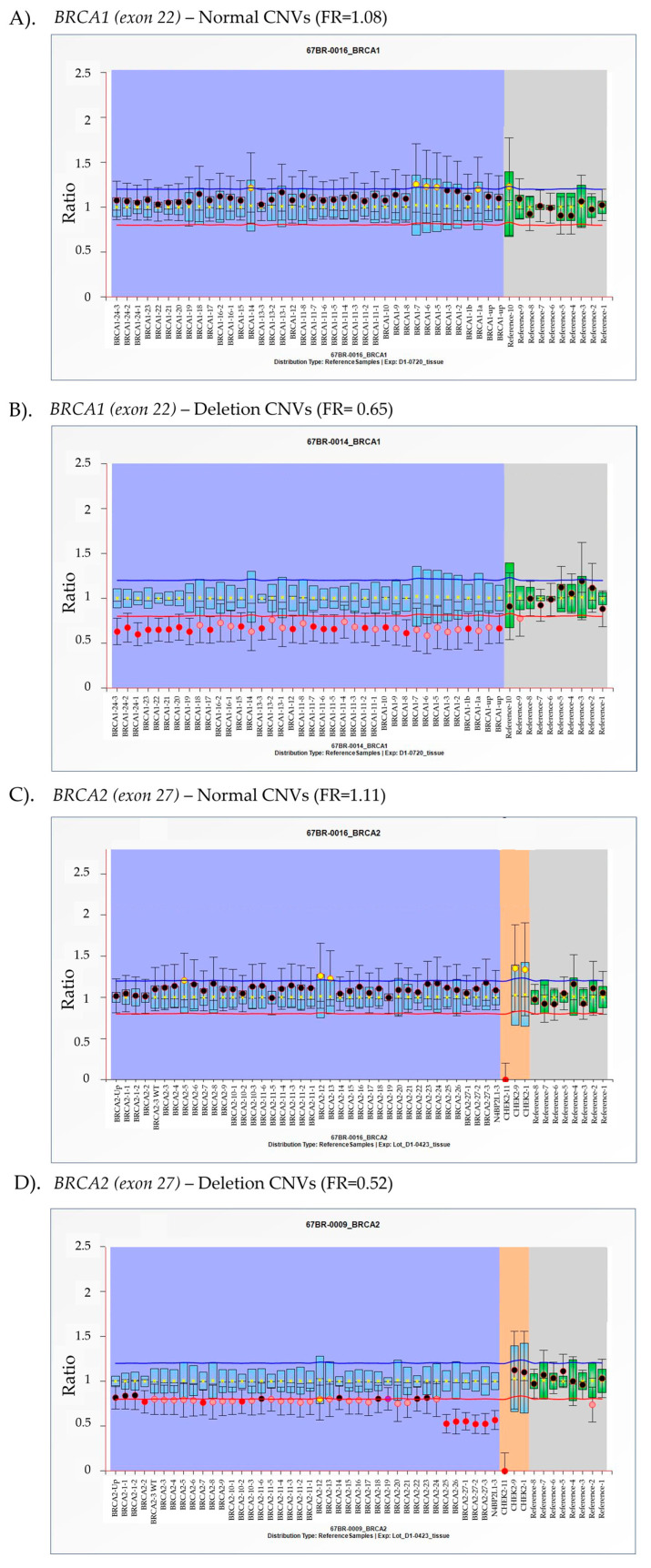
Representative multiplex ligation-dependent probe amplification (MLPA) analysis of *BRCA1/2* copy number variants (CNVs) in tissue samples from advanced prostate cancer (APC) patients. *BRCA1* (**A**,**B**) and *BRCA2* (**C**,**D**) exons were analyzed using MLPA to determine CNV status. The final ratio (FR), calculated as the fluorescence intensity of the test sample divided by the fluorescence intensity of the reference sample, is plotted on the *y*-axis, while individual exons are represented on the *x*-axis. The purple and gray areas represent the targeted gene and reference gene detection, respectively. The orange area exhibits the signal from *CHEK2* gene detection. Arbitrary thresholds were defined using the average reference sample for the same probe. FR between 0.8 and 1.2 is considered within the normal range, indicating a standard copy number. Values in the range of 0.4–0.65 suggest heterozygous deletion, while values between 0.65 and 0.80 indicate ambiguous results.

*BRCA1 exon 22* and *BRCA2 exon 27* have been reported in advance prostate cancer pathogenesis due to homologous recombinant repair defects [3,4]. A genetic alteration is CNVs, which can impact the function of both genes [21]. To investigate CNVs in DNA extracted from tissue samples, MLPA was initially performed using DNA from 11 APC tissue samples, using probe-specific amplification to detect all exons. The MLPA kit from MRC Holland©, SALSA^®^ MLPA^®^ Probe mix P002-D1 *BRCA1*, and SALSA^®^ MLPA^®^ Probe mix P045-D1 *BRCA2/CHEK2* (MRC Holland, Amsterdam, The Netherlands), were used to assess exon coverage of *BRCA1/2* CNVs. MLPA analysis of *BRCA1* and *BRCA2* was performed using probe sets corresponding to GenBank NG_005905.2 and NG_012772.3. For *BRCA1*, 48 probes produced amplification products ranging from 130 to 469 nucleotides, with 38 probes ensuring full exon coverage (exons 1–2). For *BRCA2*, 40 probes targeted the *BRCA2* gene region, while 3 probes covered *CHEK2*, collectively spanning exons 1–27. Each probe hybridized to a specific target DNA sequence and was ligated prior to amplification. Product size variations were quantified using capillary electrophoresis, which provided specific exon identification and generated fluorescence intensity as a measure of product quantity. MLPA results were expressed as final ratio values, determined by calculating the fluorescence intensity ratio of test samples relative to reference samples for each labeled exon. MLPA results were interpreted based on the final ratio, calculated by dividing the fluorescence intensity of the test sample by the reference samples. The final ratio of 0.8–1.2 was classified as a normal copy number, while a ratio below 0.8 indicated deletion. Specifically, values ranging from 0.4 to 0.65 were classified as heterozygous deletions, while those between 0.65 and 0.8 were considered ambiguous results. Representative MLPA results showing overall *BRCA1* and *BRCA2* copy number variations, including classifications of normal and deletion states, are presented in Figure 1. Pathogenic mutations in exon 22 of *BRCA1* and exon 27 of *BRCA2* disrupt DNA repair mechanisms, leading to genomic instability and increased risk of cancer and progression [3,4,22]. These alterations also influence treatment strategies, particularly the effectiveness of targeted therapies such as PARP inhibitors [23]. MLPA analysis was conducted to assess copy number changes in these key exons, providing insights into their clinical relevance.

**Figure 2:**
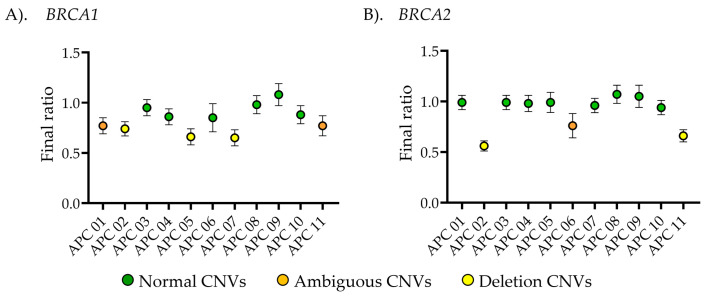
Multiplex ligation-dependent probe amplification (MLPA) detection of copy number variations in DNA extract from advanced prostate cancer (APC) patient tissues (*n* = 11). (**A**) Copy number analysis of exon 22 in *BRCA1* and (**B**) exon 27 in *BRCA2*. The *x*-axis represents the final ratio from MLPA detection, while the *y*-axis corresponds to the sample names. The center of dots represent the average final ratio, with error bars indicating the standard deviation (S.D.). Green, orange, and yellow colors represented the normal CNVs, ambiguous CNVs, and deletion CNVs, respectively.

In this study, exon 22 of *BRCA1* and exon 27 of *BRCA2* were identified as key regions for determining CNVs using MLPA. As shown in Figure 2A, six APC DNA samples exhibited a final ratio within the normal range, while three cases displayed heterozygous deletions. Additionally, two cases had FR of 0.77, indicating ambiguous results in *BRCA1* gene. For *BRCA2* CNV analysis, eight samples showed normal copy numbers, two cases exhibited deletions, and one was classified as an ambiguous result with a final ratio of 0.76 within this study group (Figure 2B). Ambiguous results were attributed to intertumoral DNA heterogeneity, characterized by the presence of multiple genetically distinct subclonal populations within a single tumor specimen. As MLPA quantifies total DNA extracted from bulk tissue, genomic alterations confined to minor subclones may be diluted by the dominant clone, resulting in weak or ambiguous signal intensities. Consequently, subclonal aberrations may fall below the detection threshold or appear inconsistent, particularly in highly heterogeneous tumors [16,24]. In these cases, tumor purity ranged from 33 to 50%, consistent with the previous studies that show that purities below 50% lead to ambiguous outcomes [16]. To ensure accurate CNV interpretation, samples with ambiguous results should be further validated or confirmed using more sensitive techniques, such as ddPCR, which can clarify the interpretation [25].

### 2.2. BRCA1/2 Determination Using ddPCR

**Figure 3:**
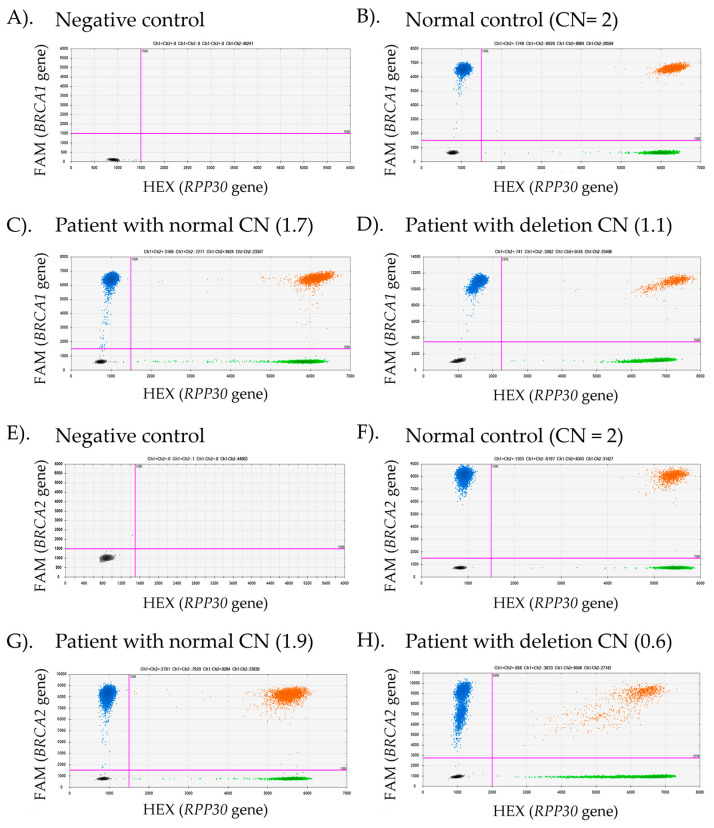
Representative droplet digital PCR (ddPCR) results for *BRCA1* and *BRCA2* copy number variant (CNV) detection in advanced prostate cancer (APC) tissues. Scatter plots illustrate fluorescence signals from two channels: FAM (blue), which detects the target genes (*BRCA1* or *BRCA2*), and HEX (green), which detects the reference gene (*RPP30*). The threshold distinguishing negative from positive droplets (indicated by pink lines) was set at one-third the average fluorescence intensity between the low- and high-intensity populations. Negative droplets appear in black, while double-positive droplets are shown in orange. The number of droplets in each group corresponds to the respective fluorescence color. (**A**,**E**) and (**B**,**F**) represent the negative control (no template control) and normal control (CN = 2), respectively. (**C**,**G**) displayed APC patient samples with normal CNVs, whereas (**D**,**H**) show APC cases with CNV deletions.

To clarify ambiguous MLPA results, we proposed the use of ddPCR, a high-throughput technology that enables the detection of small amounts of DNA and quantifies a low initial concentration of target DNA CNVs within droplet partitions. This method offers high sensitivity and reliability, making it effective for detecting small portions of CNVs in heterogeneous tissue [26]. In this study, we performed ddPCR to quantify the CNVs of *BRCA1* and *BRCA2* in the same exons as detected by MLPA, aiming to clarify the limitations in detecting DNA tumor heterogeneity, particularly in cases with ambiguous results. This preliminary investigation focuses on improving *BRCA1/2* CNV detection in tissue samples and providing insights into its clinical application. In this study, CNV detection was conducted using commercial assays from Bio-Rad, Hercules, CA, USA, including the *BRCA1* copy number assay (dHsaCP2500367), the *BRCA2* copy number assay (dHsaCP2500368), and the *RPP30* reference assay (dHsaCP2500350), which served as an internal control for data normalization. Representative ddPCR analysis results are shown in Figure 3, where fluorescence signals from both the target and reference genes are detected in each droplet partition. The fluorescence intensity varies depending on primer-probe binding to the DNA template. The absence of fluorescence signals in both the FAM-target and HEX-reference channels indicates a lack of template DNA in the ddPCR reaction (Figure 3A,E). Positive and negative droplets were classified using a fluorescence intensity threshold set at one-third of the high-intensity population in each primer-probe set. An equal or near-equivalent number of positive droplets for the target and reference genes signifies a normal copy number (CNVs ≈ 2), as shown in Figure 3B,C,F,G. A lower target copy number relative to the reference fluorescence intensity indicates a deletion, as observed in Figure 3D,H.

**Figure 4:**
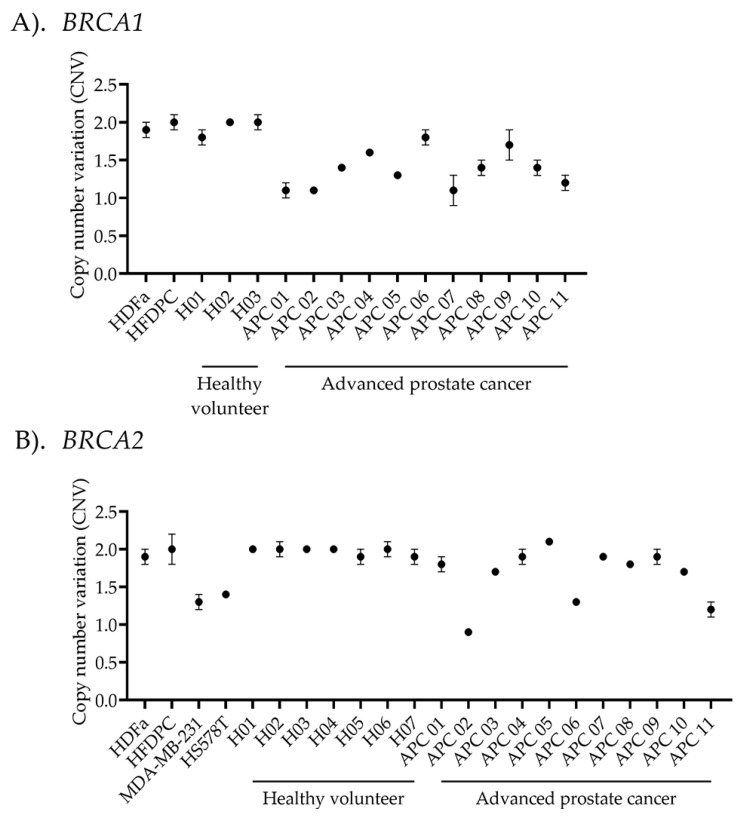
Copy number variant (CNV) analysis using droplet digital PCR (ddPCR). The plot illustrates the average ratio between (**A**) *BRCA1*- or (**B**) *BRCA2*-targeted genes and the reference gene, multiplied by diploid alleles. The calculated copy number variation (CNV) is displayed along the *x*-axis. Human dermal fibroblasts (HDFa) and human hair follicle dermal papilla cells (HFDPCs) were used as normal controls in the experiment. While MDA-MB-231 and HS578T were used as deletion controls for *BRCA2* CNV detection. CNV analysis was performed on 7 healthy volunteers and 11 advanced prostate cancer patients to detect variants in *BRCA1/2* copy numbers. Data are presented as the mean ± standard deviation (S.D.) for each sample.

For each ddPCR experiment, known copy number samples were analyzed alongside specimens from both healthy individuals and advanced prostate cancer patients to ensure quality control in every assay. As shown in Figure 4, primary dermal fibroblast (HDFa) and human hair follicle dermal papilla cells (HFDPCs) were used as normal copy number controls for the detection of *BRCA1* and *BRCA2* CNVs. Additionally, HS578T and MDA-MB-231 cell lines were used as deletion controls for *BRCA2* CNVs detection [27]. The *BRCA1/2* CNV profiles in these cell lines were obtained from DepMap portal https://depmap.org/portal (assessed on 13 May 2025). *BRCA1/2* CNV detection was performed on DNA samples from 7 healthy individuals and 11 advanced prostate cancer patients. All healthy volunteers exhibited normal *BRCA1/2* CNVs, consistent with the normal control cells. Among cancer cases performed using MLPA, six cases of *BRCA1* showed CNV values matching ddPCR results (CNVs = 1.6 ± 0.1), while eight cases of *BRCA2* detection matched with ddPCR results (CNVs = 1.9 ± 0.1). In contrast, the deletion copy number results were consistent between MLPA and ddPCR, with three cases showing *BRCA1* deletions (CNVs = 1.2 ± 0.1) and two cases exhibiting *BRCA2* deletions (CNVs = 1.1 ± 0.2). However, ambiguous results from MLPA were observed in two cases of *BRCA1* detection (CNVs = 1.2 ± 0.1) and one case of *BRCA2* detection (CNVs = 1.3 ± 0.04).

### 2.3. Correlation Between MLPA and ddPCR Results

**Figure 5:**
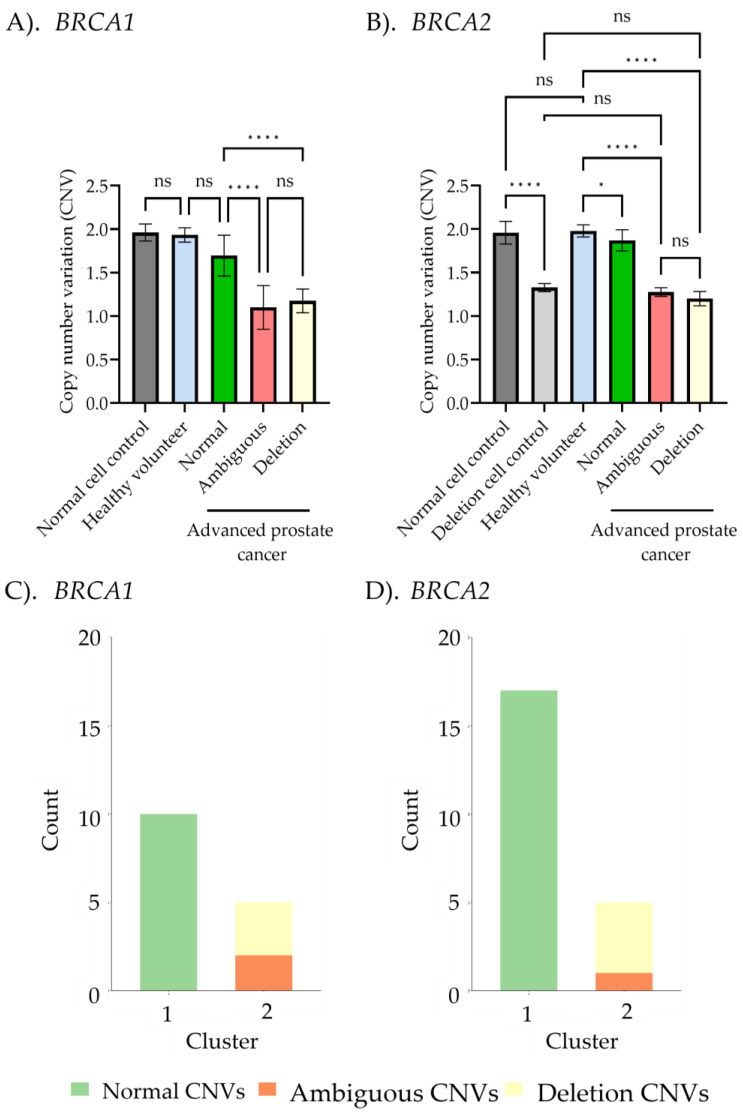
Correlation between *BRCA 1/2* copy number variant (CNV) detection using multiplex ligation-dependent probe amplification (MLPA) and droplet digital PCR (ddPCR). (**A**,**B**) Comparison of CNVs among normal controls, deletion controls, healthy controls, and the advanced prostate cancer subgroup, based on MLPA interpretation (normal CNVs, ambiguous CNVs, and deletion CNVs). Data was presented as the mean ± standard deviation (S.D.), with statistical analysis performed using one-way ANOVA followed by post hoc analysis (ns, non-significant; * p < 0.05; **** p < 0.0001). (**C**,**D**) K-means clustering from the copy number *BRCA1/2* ratios obtained from ddPCR. Green, orange, and yellow colors represent normal CNVs, ambiguous CNVs, and deletion CNVs, respectively.

Following MLPA and ddPCR analyses, we evaluated the correlation of CNV detection of *BRCA1/2* between the two methods across different sample groups, classified based on MLPA results, as presented in Figure 5. The amplification regions of *BRCA1* and *BRCA2*, obtained from both techniques and aligned with the NCBI database, are shown in Figure S1. For *BRCA1* CNV detection by ddPCR, the normal cells control, healthy volunteers, and advanced prostate cancer patients with normal final ratios from MLPA exhibited values of 2.0 ± 0.1, 1.9 ± 0.1, and 1.6 ± 0.2, respectively, with no significant differences among these groups. Interestingly, significant differences were observed when comparing these groups to the ambiguous (1.1 ± 0.2) and deletion (1.2 ± 0.1) groups in advanced prostate cancer patients. No significant differences were found between the ambiguous and deletion groups for both *BRCA1/2*. Similarly, *BRCA2* CNV detection followed the same pattern: the normal control (CNVs = 1.9 ± 0.1), healthy volunteers (CNVs = 2.0 ± 0.1), and advanced prostate cancer patients with normal CNVs (CNVs = 1.9 ± 0.1) were significantly different from the deletion cells control (CNVs = 1.3 ± 0.1), ambiguous group (CNVs = 1.3 ± 0.04), and the advanced prostate cancer group with deletion CNVs (CNVs = 1.1 ± 0.2). The CNV values obtained from ddPCR analysis in advanced prostate cancer patient groups exhibited significant two-cluster groups between normal and deletion for both *BRCA1* and *BRCA2*, as shown in Figure 5C,D. This finding demonstrates that ddPCR can effectively classify *BRCA1/2* CNVs into two groups based on CNVs identified through K-means clustering.

**Figure 6:**
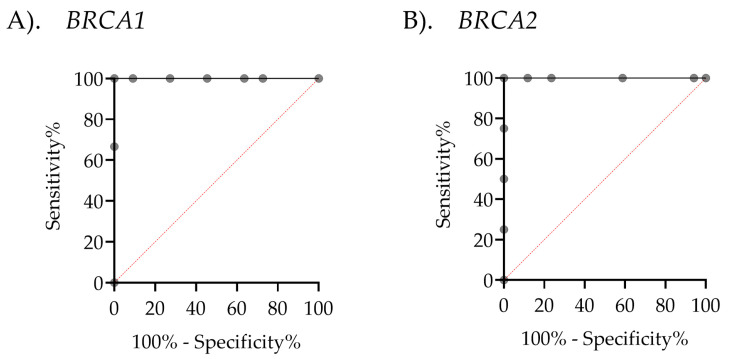
Receiver Operating Characteristic (ROC) curves assessing the performance of droplet digital PCR (ddPCR). (**A**) The ROC curve for *BRCA1* demonstrates an area under the curve (AUC) of 1, with a cutoff value of 1.350, 100% sensitivity (95% CI: 43.85–100%), and 100% specificity (95% CI: 74.12–100%). (**B**) The ROC curve for *BRCA2* shows an AUC of 1, with a cutoff value of 1.55, 100% sensitivity (95% CI: 51.01–100%), and 100% specificity (95% CI: 81.57–100%).

**Figure 7:**
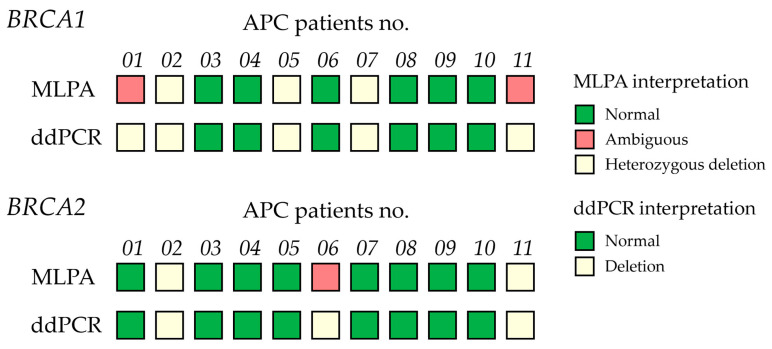
Comparison of *BRCA1/2* copy number variants (CNVs) in 11 advanced prostate cancer (APC) patient samples determined by multiplex ligation-dependent probe amplification (MLPA) and ddPCR. Concordance of MLPA and ddPCR results across samples. MLPA classification includes three groups: normal (green), ambiguous (orange), and heterozygous deletion (light yellow). ddPCR interpretation defines deletion (light yellow) as CNVs < 1.35 (*BRCA1*) and CNVs < 1.55 (*BRCA2*).

Given the clinical importance of accurately classifying CNVs, determining a precise cutoff value is essential for reliable diagnosis and decision-making. To achieve this, the Youden Index was calculated from the ROC curve across the entire sample set, excluding ambiguous results, to distinguish between normal and deletion CNVs in both *BRCA1* and *BRCA2*. This method identified the threshold that maximizes both sensitivity and specificity, ensuring precise discrimination between normal and deletion CNVs. The ROC analysis showed that the area under the curve (AUC) was 1, indicating perfect classification. The optimal cutoff CNV values were determined to be 1.35 for *BRCA1* and 1.55 for *BRCA2*, each achieving 100% sensitivity and 100% specificity (Figure 6). In the MLPA-based ambiguous group, these cutoff values enabled classification of ambiguous cases as deletions for both *BRCA1* (two cases) and *BRCA2* (one case), based on their values falling below the determined cutoff threshold (Figure 7). ddPCR is an alternative technique for detecting *BRCA1/2* CNVs in tissue DNA, overcoming the limitations of MLPA detection. For ddPCR settings, the known CNVs should be used to establish cutoff values, calculated via the Youden index in AUC, to enhance the interpretation of deletion, normal, and amplification states.

## 3. Discussion

*BRCA1* and *BRCA2* have emerged as critical biomarkers in advanced prostate cancer, given their essential roles in homologous recombination repair and genomic stability [5,6]. Alterations in these genes not only contribute to tumor progression but also influence therapy, particularly in the context of PARP inhibitors and platinum-based chemotherapy [4,10,11,12,13,17]. Given their involvement in DNA damage response pathways, assessing *BRCA1/2* CNVs provides valuable insights into disease severity and treatment resistance. Consequently, detecting CNV deletions of exon 22 in *BRCA1* and exon 27 in *BRCA2* is critical for guiding therapeutic strategies for advanced prostate cancer [4,11,17]. The ClinVar database has classified exon 22 in *BRCA2* as a likely pathogenic variant, as it generates a nuclear localization signal and an early translation stop, truncating the TR2/RAD51-binding domain [28,29]. Therefore, accurate detection of *BRCA1/2* deletions is essential for optimizing treatment strategies and improving patient outcomes.

Currently, various techniques are available for detecting *BRCA1/2* CNVs, including array comparative genomic hybridization (aCGH), as well as quantitative PCR, NGS, and MLPA [30]. Among these, MLPA is widely regarded as an efficient molecular method due to its high sensitivity, specificity, and ability to provide exon-level resolution for CNV analysis, particularly in germline specimens [30]. However, somatic CNV detection faces challenges, including potential false-positive results due to SNVs within MLPA probe annealing regions [31]. DNA quality, tissue source, and extraction methods significantly influence test performance [10,16]. Even though MLPA has been considered a gold standard tool for detecting CNVs and comparing patient DNA quantities with a control [10,16], it has certain limitations. MLPA, while a robust tool for CNV detection, may fail to identify mosaicism, leading to negative or ambiguous results when normal cells are mixed with cancer cells [9,10,16,32]. To minimize the heterogeneity-related tissues, MLPA should be performed on cancer and healthy adjacent tissue DNA from the same source [9,10,16]. In this study, MLPA produced ambiguous results in 2 of 11 cases (18.1%) for *BRCA1* and 1 case for *BRCA2*, as shown in Figure 2. High contamination by benign cells, tumor heterogeneity, and the limited sensitivity of MLPA contribute to a reduced detection rate of positive CNVs, often resulting in ambiguous findings [16]. Ambiguous results in cancer tissues present a challenge that should be clarified using alternative techniques such as targeted sequencing [33,34], real-time RT-PCR [35], or droplet digital PCR [25,36].

Droplet digital PCR (ddPCR) is a method with very high sensitivity and specificity for detecting *BRCA1/2* CNV deletions, especially in small DNA concentrations, and it offers several advantages over MLPA [19,30]. As a next-generation PCR technique, ddPCR enables absolute quantification of nucleic acids without requiring a standard curve. This approach provides superior sensitivity, specificity, and precision, particularly in detecting small fold changes or heterozygous deletions in mosaic tissue samples [37,38]. To address the challenges of CNV detection in such samples, we applied ddPCR to analyze *BRCA1* exon 22 and *BRCA2* exon 27 using DNA derived from both blood and frozen tumor samples. These exons were selected based on previous reports linking *BRCA1/2* alterations with sensitivity to PARP inhibitors, as well as their relevance in CNV detection models derived from ovarian cancer cell lines, which are commonly used to study therapeutic targets and molecular mechanisms of disease progression [39,40,41,42,43]. In ddPCR, the number of droplets defines the dynamic range of target DNA quantitation. While MLPA requires highly trained experts and extensive bioinformatics analysis for *BRCA1* and *BRCA2* interpretation [9,10], ddPCR provides an absolute measurement of DNA abundance by measuring the DNA copy number in each droplet partition [9,18,44].

Despite its superior sensitivity and precision, establishing a reliable ddPCR assay demands rigorous optimization and validation against gold standard methods. Crucially, the use of well-characterized normal and deletion control cell lines is essential for setting accurate cutoff thresholds [45]. In this study, normal cell controls, such as primary dermal fibroblasts and human follicle dermal papilla cells, served as the diploid status of *BRCA1/2*. Known deletion controls, including TNBC cell lines like MDA-MB-231 and HS578T, demonstrated reduced *BRCA2* copy numbers, supporting the role of copy number loss in functional BRCA deficiency in sporadic TNBCs, even without pathogenic mutations, as reported in the DepMap portal. Incorporating these controls ensures that ddPCR can distinguish subtle copy number changes, including heterozygous deletions, from normal diploid states, providing robust technical validation necessary for biomarker development and clinical application Figure 4. Several studies have demonstrated high ddPCR sensitivity of 98%, compared to MLPA, which reports a sensitivity range of 95–99% [7,9,18,29], highlighting the importance of proper validation in ddPCR assay setup.

As shown in Figure 5, CNV analysis using ddPCR demonstrates significant differences between normal copy number cases, including normal cell control, healthy volunteers, and APC samples with normal final ratios bases on MLPA interpretation and the deletion copy number group, which includes deletion cell control, APC samples with confirmed deletions, and ambiguous group (non-significant deletion). These results are consistent with previous studies that demonstrate the capacity of ddPCR to accurately detect fold changes as small as 1.2× in the gene copy number, underscoring its high sensitivity for evaluating somatic molecular alterations [37,46,47]. Notably, ddPCR has been applied to resolve ambiguous CNV classifications from MLPA in some diseases. For example, in spinal muscular atrophy (SMA), ddPCR was used to resolve ambiguous MLPA findings and showed concordance with clinical phenotypes [48]. In breast cancer, ddPCR-based detection of *HER2* CNVs has demonstrated high consistency with HER2 immunohistochemistry and in situ hybridization, achieving a concordance rate of 92.9% [44]. Given its ability to refine CNV classification, ddPCR has emerged as a highly effective technique in clinical applications, improving CNV detection and enhancing tumor diagnosis, particularly in tissue DNA samples. This is especially relevant for *BRCA1/2* CNV detection, where precise genomic insights are crucial for enabling clinicians and patients to make timely, targeted treatment decisions [9,49]. Additionally, ddPCR requires only minimal amounts of DNA and is well suited for amplifying short fragment size (<100 bp), enabling the detection of key genomic alterations in various types of cancers and sample sources. For example, ddPCR has been successfully applied to detect *HER2* CNVs in formalin-fixed, paraffin-embedded (FFPE) breast carcinoma tissue, *BRCA1* CNVs in whole blood-derived breast and ovarian cancers, *MYCN* amplification in neuroblastoma FFPE samples, *MET* CNVs in FFPE gestic cancer, and hepatocellular carcinoma. It has also been used to detect complex CNVs such as *JAK1-PAK2*, *JAK1-PVT1*, *JAK1-MYOCD*, *JAK1-TIMM21*, *USP7-TIMM21*, and *JAK1-Chr22* CNVs in whole blood-derived ovarian cancer samples, *EGFR*, *CDKN2A*, and *BRAF* CNV in glioma tissues, as well as *FGFR2* CNV in colorectal and gastric adenocarcinomas [19,26,50,51,52,53,54,55]. Moreover, *BRCA1/2* CNV detection using ddPCR has been performed to investigate high-grade serous ovarian cancer patient-derived cell line models [43]. Interestingly, the *BRCA1/2* CNV detection in FFPE APC tissues using ddPCR has not yet been reported. These advantages address common challenges associated with FFPE tumor testing and further underline ddPCR’s clinical utility.

To our knowledge, *BRCA1/2* CNV detection using droplet digital PCR (ddPCR) in advanced prostate cancer (APC) tissue has not been previously reported. This study is the first to demonstrate that ddPCR, when calibrated with well-characterized reference controls, can effectively resolve ambiguous MLPA results in APC samples. These findings underscore the clinical potential of ddPCR to improve diagnostic accuracy and inform therapeutic decision-making in precision oncology. Our study therefore represents a meaningful contribution, highlighting both the feasibility and clinical utility of ddPCR for *BRCA1/2* CNV detection in this context. Notably, ROC curve analysis (Figure 6) demonstrated 100% sensitivity and specificity for *BRCA1/2* CNV detection, highlighting the technique’s potential for clinical application in advanced prostate cancer diagnostics. Furthermore, our results are consistent with prior studies reporting robust ddPCR performance in cancer detection; for instance, an investigation into *CDKN2A* copy number variations in 57 FFPE melanoma samples yielded a sensitivity of 94.4% and a specificity of 90.0% [50]. *BRCA1* CNV detection in whole blood-derived ovarian and breast cancer sample achieved 100% concordance with MLPA, supporting that ddPCR’s comparability with the established gold standard method for CNV assessment [19]. Similarly, ddPCR also successfully distinguished *HER2* amplification levels in FFPE tumor tissues, with 100% concordance to both FISH and IHC analyses [26].

For clinical application of ddPCR detecting *BRCA1/2* CNVs in cancer tissues, a critical consideration is the establishment of appropriate cutoff thresholds to distinguish between normal and deletion classifications. Previous studies have shown that cutoff values for CNV detection using ddPCR vary depending on the target gene, specimen type, and assay conditions. This variability underscores a key limitation of ddPCR as the lack of standardized thresholds for CNV classification. Reported cutoff values typically range from 1.15 to 1.73, reflecting differences in experimental design and sample characteristics [54,56]. In this study, the optimal cutoff can be determined using specific statistical measures, such as Youden’s index [57]. This index is one of the measures of diagnostic accuracy, which selects the value that maximizes the sum of sensitivity and specificity minus one at a certain point on the ROC curve [57,58]. Previous studies have applied Youden’s index to optimize ddPCR cutoffs for detecting *Pneumocystis pneumonia* [59] and SARS-CoV-2 virus [60], as well as for applications beyond pathogen detection, such as mutation and gene expression analysis, including CNV screening for thalassemia [56], spinal muscular atrophy (*SMA*) gene assessment in SMA [61], and quantification of *Mycobacterium tuberculosis* [62]. In our study, ROC curve and Youden’s index analyses were performed using known CNV cell lines, healthy controls, and clearly interpreted APC cases identified by MLPA, as shown in Figure 6. The optimal cutoff values identified for *BRCA1* and *BRCA2* were 1.35 and 1.55, respectively. Applying these cutoffs enabled the reclassification of ambiguous MLPA results as deletions (Figure 7), consistent with Figure 5, showing that ambiguous *BRCA1/2* CNVs did not significantly differ from the deletion group. These results highlight ddPCR as a high-throughput, reliable method for clarifying *BRCA1/2* CNV status in heterogeneous tissue DNA samples. Overall, to establish clinical utility, it is essential to validate ddPCR thresholds against gold standard methods using well-characterized reference samples, incorporating Youden’s index or the approach outlined in this study.

In addition to concerns over cutoff determination and maintaining high sensitivity and specificity with limited DNA, turnaround time is a critical factor for clinical application. A streamlined and less complex approach to establish *BRCA1/2* CNV status in heterogeneous tissue samples is essential for clinical translation. The optimized workflow requires minimal DNA input and completes analysis within 8 h from DNA extraction, offering a significantly shorter turnaround time compared to other molecular amplification methods. In contrast, techniques such as FISH or MLPA typically require more than 24 h for detection and analysis [9,63]. MLPA is more time-consuming because it requires the hybridization of probes binding to specific DNA sequences (which takes approximately 16 h), a step that is eliminated in ddPCR methods [9,18].

Overall, our findings provide evidence that ddPCR is particularly well suited for clinical and translational applications due to its low DNA input requirements, robustness to sample quality, and compatibility with FFPE tissue. These features not only make ddPCR a valuable tool for confirming *BRCA1/2* alterations and potentially guiding targeted therapies in APC patients, but they also suggest its broader applicability for detecting CNVs across various clinical settings. However, significant challenges remain to be addressed before ddPCR can be routinely implemented in clinical practice. In particular, we acknowledge that the limited sample size in this study constrains the ability to draw definitive conclusions regarding its clinical superiority over multiplex ligation-dependent probe amplification (MLPA). Nevertheless, previous studies have consistently indicated that ddPCR can yield reproducible and clinically relevant results even in small cohorts ranging from 5 to 24 samples, particularly during assay optimization and feasibility assessments for CNV detection in diseases such as breast and ovarian cancer [19,25,55]. To increase robustness, further studies should be performed on larger validation cohorts (N = 200–300) [64]. Moreover, the integration of advanced computational strategies could help address challenges associated with small sample sizes and the complexity of high-dimensional genomic data. Such approaches may enhance model robustness, improve feature interpretability, and increase predictive accuracy [65,66,67]. Therefore, larger-scale studies with a broader range of CNV profiles are necessary to validate these preliminary findings.

In addition to increasing sample sizes to improve cutoff threshold determination, improving availability of well-characterized control samples, and achieving more comprehensive primer coverage, especially for detecting pathogenic variants linked to FDA-approved therapies. *BRCA1* exons 11–13 and *BRCA2* exons 11 and 27 are hotspots for pathogenic variants that encode binding domains for key proteins such as RAD51 [22,42,68]. Incorporating multiplex ddPCR assays targeting these regions may improve detection sensitivity and enable more precise patient stratification for PARP inhibitor therapy.

To ensure reproducibility and reliability, these findings should be validated through both intralaboratory and interlaboratory studies and should be performed under these experimental conditions. Each ddPCR run should achieve the key quality control criteria as generation of ≥20,000 droplets, no signal in negative droplets, co-amplification of target and reference primers within the same well, inclusion of known *BRCA1/2* CNV cell lines, and application of a consistent cutoff value for interpretation. These parameters serve as critical quality control measures to demonstrate the accuracy, precision, and interpretive reliability of ddPCR-based CNV detection. For future studies, collecting tumor tissue, adjacent normal tissue, and blood specimens from the same patients will help distinguish between somatic and germline alterations, in alignment with national APC treatment guidelines.

Importantly, ddPCR overcomes key limitations of MLPA, particularly in detecting somatic mutations within highly heterogeneous, low-percentage tumor purity samples, positioning it as a promising alternative for precision oncology biomarker detection. Additionally, complementary analytical techniques will be essential to validate ambiguous results and confirm the concordance of ddPCR findings. Ultimately, while our results highlight the potential of ddPCR for *BRCA1/2* CNV detection in advanced prostate cancer, further validation is critical before its routine clinical implementation can be recommended. Nonetheless, this study represents a meaningful advancement towards the clinical translation of ddPCR-based *BRCA1/2* CNV detection, and these integrated tools may ultimately support the development of clinically actionable CNV biomarkers in prostate cancer and other malignancies.

## 4. Materials and Methods

### 4.1. Specimen Collection

This study was approved by the Institutional review boards (IRBs) of the Faculty of Medicine, Prince of Songkla University (approval numbers: REC 64-565-25-2). All participating patients voluntarily signed an informed consent form to participate and to have their biological specimens analyzed. All procedures performed in the studies involving human participants were conducted in accordance with the ethical standards of the institutional research committee. Histopathological assessments were determined by pathologists at the Department of Pathology, Songklanagarind Hospital. Eleven patient specimens for prostate cancer were obtained, all meeting the following inclusion criteria: advanced prostate cancer, treatment with androgen deprivation therapy (ADT), surgical cancer castration, and a Gleason score equal or greater than 7. The patient underwent surgery as part of the routine treatment regime to remove cancerous tissue via transurethral resection of the prostate. To determine the normal copy number, whole blood samples were collected from healthy volunteers (n = 7). The characteristics of all patients are shown in Table S1. Following collection, red blood cells were lysed to isolate white blood cells, which were subsequently stored at −80 °C for further analysis.

### 4.2. Cell Lines

Primary human dermal fibroblasts (HDFa, PCS-201-012™), HS578T (HTB-126™), and MDA-MB-231 (HTB-26™) were obtained from American Type Culture Collection (ATCC, Manassas, VA, USA). Human hair follicle dermal papilla cells (HFDPCs) were purchased from Promocell (Heidelberg, Germany). HDFa, HS578T, and MDA-MB-231 were maintained in DMEM medium (Gibco, Paisley, UK) with 10% fetal bovine serum (Gibco, Paisley, UK), 1X GlutaMAX™ (Gibco, Paisley, UK), and 1X penicillin/streptomycin (Gibco, Paisley, UK). HFDPCs were cultured using a follicle dermal papilla cell growth medium mixed with a growth medium supplement mix (Promocell, Heidelberg, Germany). All cultures were maintained at 37 °C in a humidified incubator with 5% CO_2_. Cells were pelleted, collected, and stored at −80 °C as a control for known CNVs in ddPCR.

### 4.3. DNA Extraction

DNA was extracted using the DNeasy Blood and Tissue Kit (QIAGEN, Valencia, CA, USA) following the manufacturer’s protocol. Briefly, tissue samples (1–2 mm^2^ in size, 3–4 pieces, total weight < 25 mg), cell pellets, and white blood cells were lysed in lysis buffer containing proteinase prior to DNA purification using the DNeasy Mini spin column. DNA concentration and purity were assessed using a Nanodrop 2000 spectrophotometer (Thermo Scientific, Waltham, MA, USA), and aliquots were stored at −20 °C for downstream analyses.

### 4.4. Detection of BRCA1/2 Deletion Using Droplet Digital PCR

Droplet digital PCR was performed to quantify CNVs of *BRCA1* exon 22 (chromosome 17) and *BRCA2* exon 27 (chromosome 13), using RPP30 (chromosome 10) as the reference gene (endogenous control) for internal normalization in each sample. The CNV detection kits from Bio-Rad (Hercules, CA, USA) used were *BRCA1* (dHsaCP2500367), *BRCA2* copy number assay (dHsaCP2500368), and *RPP30* assay (dHsaCP2500350). Mapping of ddPCR target positions were indicated in Figure S1. Within each independent experiment, ddPCR included no template control (NTC), normal copy number cell control (HDFa and HFDPCs). Following the manufacturer’s instructions, the PCR reaction consisted of 2X ddPCR™ Supermix for Probes (Bio-Rad, Hercules, CA, USA), a FAM-labeled target (*BRCA1* or *BRCA2*) primer-probe mix, a HEX-labeled reference primer-probe mix, Hae III restriction enzyme (New England Biolabs, Ipswich, MA, USA), and either 10 ng of DNA template or nuclease-free water (NTC). Droplets were generated using the Bio-Rad Automated Droplet Generator, followed by thermocycling on the Bio-Rad T100 Thermal Cycler according to the manufacturer’s protocol: 95 °C for 10 min, 40 cycles at 94 °C for 30 s and 55 °C for 1 min, 98 °C for 10 min, and a final hold at 4 °C. Subsequently, all droplets were detected using the Bio-Rad QX200 Droplet Reader (Bio-Rad, Hercules, CA, USA). For quality control, the total number of droplets should be approximately 20,000. Positive and negative droplets were analyzed using QuantaSoft™ version 1.7.4. The threshold cutoff was determined as one-third between the signal intensity of negative and positive droplets. CNVs were calculated by dividing the number of positive droplets (copy number) for the target gene by the copy number for the reference gene and multiplying by two to account for diploid alleles.

### 4.5. Identifying Exon Deletion of BRCA1/2 Using MLPA

The MLPA reactions were performed in accordance with the manufacturer’s instructions. Denatured genomic DNA from tissues was hybridized with MLPA probes using the SALSA MLPA probe mix (*BRCA1*: P002; *BRCA2*: P045, MRC Holland). The reactions, including negative control samples, were conducted exactly as directed by the manufacturer. Electrophoresis was performed using the Applied Biosystems 3500 Genetic Analyzer (Thermo Fisher Scientific, Waltham, MA, USA), and analysis was carried out using Coffalyser.Net software version 220513.1739. Interpretation was based on the results from this software.

### 4.6. Statistics

All the results and graphs were statistically analyzed and generated using GraphPad Prism version 10.4.2. Normality was performed using the Shapiro–Wilk test, prior to comparing the normal, ambiguous, and deletion groups using one-way ANOVA with post hoc analysis (* *p* < 0.05, ** *p* < 0.01, *** *p* < 0.001, **** *p* < 0.0001). In addition, K-means clustering was applied to classify CNV groups based on ddPCR copy number ratios. The analysis was performed using R version 4.5.0, employing the built-in kmeans function without external packages. Parameters were set to centers = 2 and nstart = 100 to ensure robust clustering of normal, ambiguous, and deletion CNV groups. To optimize classification, the cutoff value distinguishing normal from deletion copy numbers was determined using the Youden Index. This index identifies the specific point on the ROC curve where the sum of sensitivity and specificity is maximized using the formula: (sensitivity + specificity) − 1. All calculations were performed using GraphPad Prism version 10.4.2.

## 5. Conclusions

Our study demonstrates that ddPCR provides more reliable and sensitive detection of *BRCA1/2* CNVs in advanced prostate cancer tissues compared to MLPA, especially in heterogeneous samples. Validation in larger cohorts is necessary to optimize cutoff precision and capture a wider spectrum of CNV alterations. Moreover, integration with targeted sequencing can further help validate discrepancies between ddPCR and MLPA, thereby enhancing diagnostic accuracy. Additionally, extending primer coverage to include known pathogenic variants associated with FDA-approved therapies would further augment the clinical relevance of this approach, ensuring the comprehensive detection of alterations that inform personalized treatment decisions. Overall, the high sensitivity and precision of ddPCR make it a robust platform for clinical *BRCA1/2* CNV testing, enabling improved biomarker detection and supporting personalized treatment strategies in advanced prostate cancer.

## Data Availability

Data is contained within the article and Supplementary Materials.

## Supplementary Materials

The supporting information can be downloaded at: https://www.mdpi.com/article/10.3390/ijms26146904/s1.

## Abbreviations

The following abbreviations are used in this manuscript:

CNVs	Copy number variants
MLPA	Multiplex ligation-dependent probe amplification
ddPCR	Droplet digital PCR
APC	Advanced prostate cancer
HRR	Homologous recombinant repair
PSA	Prostate-specific antigen
SNVs	Single-nucleotide variants
LGR	Large genomic alteration
PARP	Poly (ADP-ribose) polymerase
NGS	Next-generation sequencing
S.D.	Standard deviation
HDFa	Primary dermal fibroblast
HFDPCs	Human hair follicle dermal papilla cells
AUC	Area under the curve
ROC	Receiver operating characteristic
aCGH	Array comparative genomic hybridization
SMA	Spinal muscular atrophy
IRBs	Institutional review boards
ADT	Androgen deprivation therapy
