# Supplementary material for: Droplet Digital PCR Improves Detection of BRCA1/2 Copy Number Variants in Advanced Prostate Cancer
Source: Int J Mol Sci. 2025 Jul 18;26(14):6904. doi: 10.3390/ijms26146904 (PMC12295428; doi:10.3390/ijms26146904)
Supplement: Supplementary file 1 [file ijms-26-06904-s001.zip › ijms-3728841-supplementary.pdf]

A). *BRCA1*

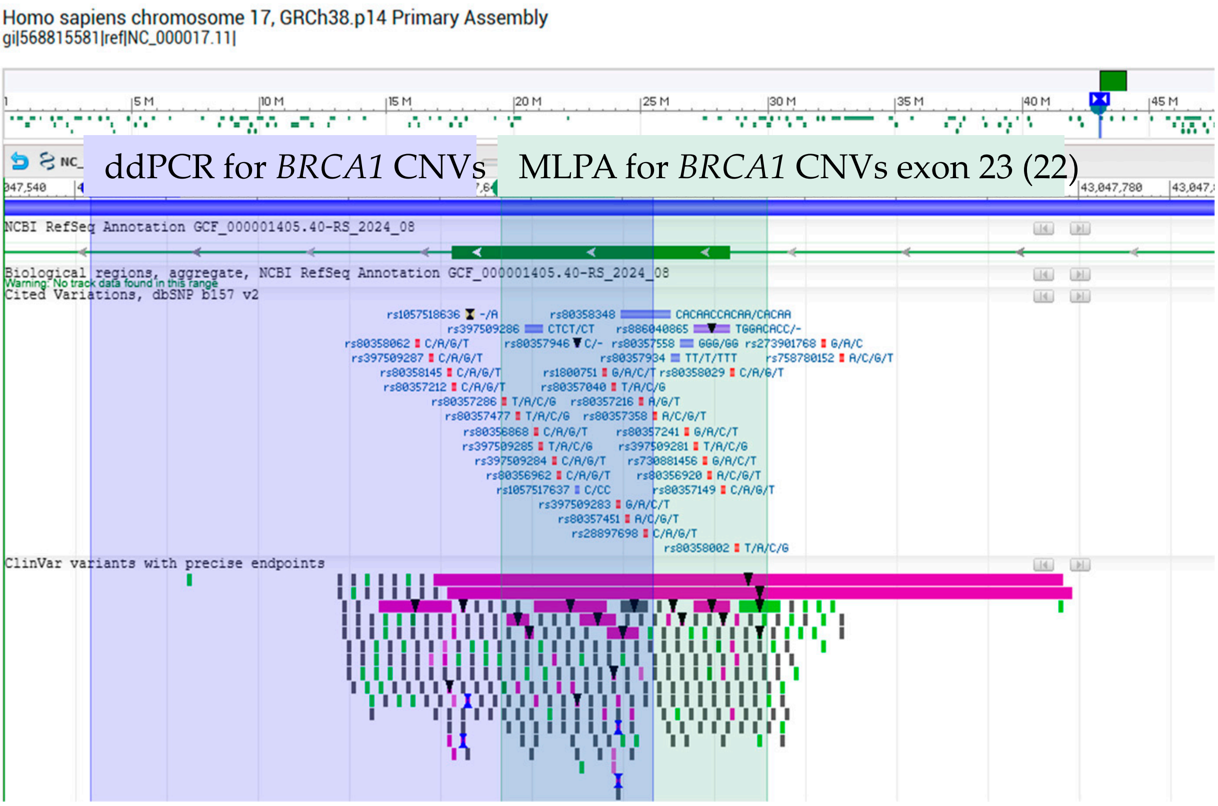

B). *BRCA2*

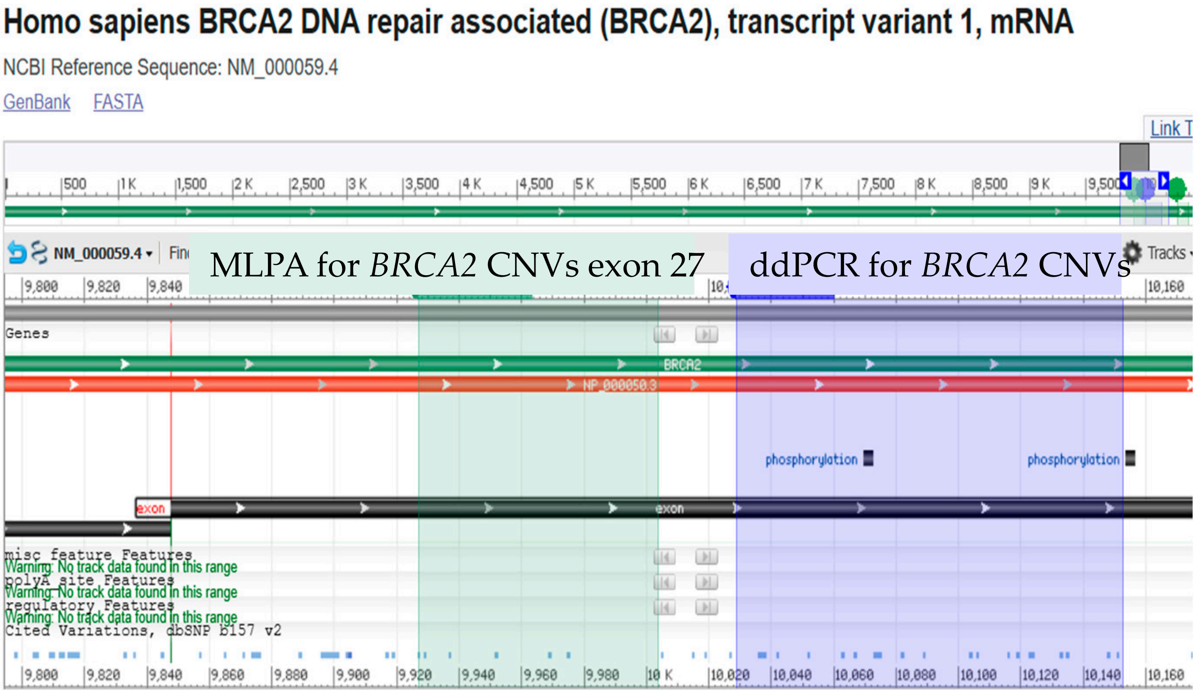

Figure S1. Mapping of MLPA and ddPCR target positions in NCBI BLAST for the BRCA1 gene (NCBI Reference Sequence: NC_000017.11) and BRCA2 gene (NCBI Reference Sequence: NM_000059.4).

Table S1. Characteristics of patients

	Healthy individuals	Advance prostate cancer
Number of patients (N)	8	11
Median age, year (Median±IQR)	64±21.5 (54-89)	70±10.5 (55-90)
Gleason score at diagnosis (IQR)		8±2 (7-9)
Medium PSA, ng/ml (IQR)		75.4±218.3 (26.6-611)
Androgen deprivation therapy (ADT)		Yes
